# The use of cellulolytic *Aspergillus* sp. inoculum to improve the quality of Pineapple compost

**DOI:** 10.3934/microbiol.2023003

**Published:** 2023-02-07

**Authors:** Bambang Irawan, Aandi Saputra, Salman Farisi, Yulianty Yulianty, Sri Wahyuningsih, Noviany Noviany, Yandri Yandri, Sutopo Hadi

**Affiliations:** 1 Department of Biology, Faculty of Mathematics and Natural Sciences, the University of Lampung, Bandar Lampung, Lampung, Indonesia; 2 Department of Chemistry, Faculty of Mathematics and Natural Sciences, the University of Lampung, Bandar Lampung, Lampung, Indonesia

**Keywords:** *Aspergillus* sp, cellulolytic, decomposition inoculum, pineapple litter

## Abstract

Pineapple litter has a complex polymer of cellulose, hemicellulose, and lignin, which makes them difficult to decompose. However, pineapple litter has great potential to be a good organic material source for the soil when completely decomposed. The addition of inoculants can facilitate the composting process. This study investigated whether the addition of cellulolytic fungi inoculants to pineapple litters improves the efficiency of the composting processes. The treatments were KP1 = pineapple leaf litter: cow manure (2:1), KP2 = pineapple stem litter: cow manure (2:1), KP3 = pineapple leaf litter: pineapple stem litter: cow manure P1 (leaf litter and 1% inoculum), P2 (stem litter and 1% inoculum), and P3 (leaf + stem litters and 1% inoculum). The result showed that the number of *Aspergillus* sp. spores on corn media was 5.64 x 10^7^ spores/mL, with viability of 98.58%. *Aspergillus* sp. inoculum improved the quality of pineapple litter compost, based on the enhanced contents of C, N, P, K, and the C/N ratio, during the seven weeks of composting. Moreover, the best treatment observed in this study was P1. The C/N ratios of compost at P1, P2, and P3 were within the recommended range of organic fertilizer which was 15–25%, with a Carbon/Nitrogen proportion of 11.3%, 11.8%, and 12.4% (P1, P2, and P3), respectively.

## 1. Introduction

Lignocellulosic biomass is the most abundant biological material on earth. An important fraction of lignocellulosic biomass corresponds to waste from different economic activities, particularly, agricultural and agro-industrial residues such as cereal straws, corn stover, coffee husks, coconut fiber, wood, and forestry waste, palm press fiber, and palm kernel shells, among others [1]. One of the lignocellulosic biomass sources is pineapple plantation residue. Pineapple leaves contain a high fiber content, including cellulose, hemicellulose, and lignin at 43.53%, 21.88%, and 13.88%, respectively [2]. Also, the content of pineapple humps was cellulose, hemicellulose, and lignin at 24.53%, 28.53%, and 5.78%, respectively [2]. These chemical contents are polymers that are difficult to decompose. The common practice of clearing pineapple (*Ananas comosus*) residues for land preparation for cultivation is by burning and unsustainable agricultural practice that causes environmental pollution. Besides, the disposal of this waste implies high costs and negative environmental impacts. As a source of valuable sugars and polymers, lignocellulosic biomass can be used for the production of a broad spectrum of chemicals and materials such as liquid, gaseous, and solid biofuels, enzymes, organic compounds, synthetic polymers, pharmaceuticals, and food products, among many others [1]. In particular, pineapple wastes may be employed for producing high-quality organic fertilizer [3].

The main obstacle to the natural decomposition of the lignocellulosic residues from agricultural, agro-industrial, and forestry activities is their high content of lignin in the lignocellulosic complex bound to cellulose and hemicellulose. Also, in pineapple waste, cellulose components are not easily degraded, either chemically or mechanically, because cellulose has crystalline properties and is insoluble, which comes from its linear structure [4].

Biodegradation of biomass is carried out by different heterotrophic microorganisms, bacteria, fungi, actinomycetes and protozoa [5]. The decomposition process of degradable substrates containing cellulose, hemicellulose and lignin indicates that fungi are the microorganisms playing a major role instead of bacteria [6]. In order to degrade pineapple litters, biological steps are likely to be carried out with the help of enzymes being produced by microorganisms [7],[8]. One of the microorganisms speeding up the decomposition process is the fungus group. Fungi excrete enzymes that degrade carbohydrate polymers into simple compounds and release reducing sugars (glucose) as the final product, with reduced glucose being an essential nutrient for the microorganism's survival. Fungi are also the main degrading microorganisms of organic material in the natural environment, as they utilize insoluble compounds, such as cellulose and lignin [7],[8].

Applying fungal inoculums in the composting process is intended to accelerate the conversion of complex polymeric of pineapple litter into simple elements, which are returned to the soil as mineral nutrients. The ability of fungi to hydrolyze cellulose is carried out through the cellulase activity it possesses. The fungus groups remodeling cellulose with the help of cellulase enzymes are called cellulolytic fungi [9]. Previous studies reported that some fungal genera containing cellulolytic abilities include genera *Aspergillus, Penicillium*, and *Paecilomyces* [10]. Moreover, *Helminthosporium* sp., *Cladosporium* sp., *Trichoderma* sp. and *Aspergillus* sp. were screened for their highest cellulolytic enzyme activity [11]. Also, some *Aspergillus* sp. reported to have the potential cellulolytic ability are *Aspergillus niger* [12], *A. fumigatus* [13], *A. aculeatus* [14]. Hypothetically, the addition of a cellulolytic *Aspergillus* sp. in composting pineapple biomass which is rich in complex organic compounds, will degrade these compounds into simple monomers and release many nutrients for plants.

Based on its contents, pineapple litter has great potential as an organic material, when perfectly decomposed. This research is related to the efforts in accelerating and improving the quality of organic material decomposition. The time and quality of pineapple litter decomposition are also maximized by inoculum fungi, *Aspergillus* sp. The inoculum containing a pure culture of *Aspergillus* sp. are likely to be created, using various substrates. Corn has a high cellulose content, which is suitable as a cellulolytic fungi growth substrate, with the inoculum then applied to the pineapple litter. The addition of cellulolytic fungi inocula plays a role in litter composting, due to having the best enzymatic ability to decompose, while also producing high-quality compost [15]. Corn is used as an inoculum medium for *Aspergillus* sp. because it is rich in cellulose, which is a suitable substrate for the growth of cellulolytic isolates It is expected to increase the spore number and high viability. In this study, we used different parts of pineapple litter and these parts were chopped into a mixture.

In order to discover the quality of *Aspergillus* sp. as an inducer of cellulose decomposition, the preparation of the cellulolytic fungi (*Aspergillus* sp.) with corn media and its effect on pineapple litter composting was investigated.

## 2. Materials and methods

### 2.1. Materials and analysis

The isolate of *Aspergillus* sp. (Bioggp 3) was obtained from a previous study in which they were isolated from the isolation process of mixed leaf litter and soil taken from the pineapple plantation of PT. Great Giant Pineapple (PT. GGP) Terbanggi Besar, Central Lampung, Indonesia. The isolate showed cellulolytic and xylanolytic activity with a cellulolytic index 4.00 ± 0.783 and xylanolytic index 4.20 ± 1.03 respectively [16]. The compost material used is pineapple plant biomass which consists of leaves and stems. The selection of cellulolytic fungi isolates was done by modification of the Congo-Red method [17]. Isolates were obtained were cultured in Cellulose Agar (cellulose 5.0, NaNO_3_ 1.0, K_2_HPO_4_ 1.8, MgSO_4_.7H_2_O 0.9, KCl 0.5, 0.5 yeast extract, casein hydrolysat 0.5, agar 20 and distilled water 1L). Confirmation of cellulose-degrading ability of fungal isolates was performed by streaking it on cellulose agar media. Media were 2 layer media (bilayer) with the bottom layer was a PDA of 1/5 recipes, agar 1.5, and distilled water 100 mL. The top layer was Carboxymethyl Cellulose (CMC) 1–2%, agar 1.5 and distilled water 100 mL. Once inoculated with fungi in the middle of the test media, the cultures were then incubated for 4 days [18]. The media were added with 0.1% Congo-Red and allowed to stand for 20 minutes at room temperature. Media was washed with 1 M NaCl. Isolates producing cellulase formed a halo (clear zone) around the colony. The use of Congo-Red as an indicator for cellulose degradation in an agar medium provides the basis for a rapid and sensitive screening test for cellulolytic fungi. Colonies showing decolorization of Congo-Red were taken as positive cellulose-degrading fungal colonies [7], and only these were taken for further study. The production of *Aspergillus* sp. inoculum was carried out in the Microbiology Lab, while composting applications were conducted at the Green House Botany Laboratory, Department of Biology, Universitas Lampung. Compost chemical analysis was carried out at PT. GGP.

### 2.2. Procedure

The research used a single factor Completely Randomized Design (CRD), as a treatment that is the difference in the composition of compost materials arranged in 6 levels: KP1; KP2; KP3; P1; P2, and P3. Each treatment was carried out in 3 repetitions; hence it obtained 18 experimental units [19].

The stages involved were:

(1) Inoculum development,

(2) Inoculum application in pineapple litter composting.

Inoculum development was made using modification of Gaind et al. method [20]. Corn grains were used as substitute for fungal strain growth. The corn grains were finely ground and sifted before it was mixed with 4% calcium sulphate, and 2% calcium carbonate (in 1 L distilled water). A loopful of *Aspergillus* sp. culture was inoculated in each 100 g corn grains added with 25 mL of solutions (sterilized at 15 lb pressure for 1 h) and incubated at 25 °C for 15 days. Each strain's whole growth, including mycelium, spores, and the grains, was used as the inoculum. The inoculum was counted for the number of spores and viability by calculating CFUs [18].

Composting was carried out by modifying the Takakura Home Method (THM) [21], for 7 weeks. The composting process was carried out in a perforated basket with a lid. Basket was lined with cardboard to keep the conditions moist when composting. Next, compost materials were put in the basket and add with *Aspergillus* sp inoculum.

The composition of raw materials were pineapple leaf, stem litters and mixture of both created into 6 treatments (KP1, KP2, KP3, P1, P2, and P3; K = treatments without inoculum), as the following details,

KP1 = pineapple leaf litter:cow manure (2:1)

KP2 = pineapple stem litter:cow manure (2:1)

KP3 = pineapple leaf litter:pineapple stem litter:cow manure (1:1:1)

P1 = pineapple leaf litter:cow manure (2:1) + 1% inoculum (30 g)

P2 = pineapple stem litter:cow manure (2:1) + 1% inoculum (30 g)

P3 = pineapple leaf litter: pineapple stem litter: cow manure (1:1:1) + 1% inoculum (30 g)

The compost quality testing was carried out by analyzing the levels of carbon (C), nitrogen (N), phosphorus (P), potassium (K), and C/N ratio. Total organic carbon was determined using wet digestion method [22]. Nitrogen totals were calculated by the Kjeldahl method [23]. Phosphorus was measured by a spectrophotometer using phosphomolybdate blue method [24]. Potassium was measured by a flame photometer.

### 2.3. Variable observation and data analysis

The parameters measured in this study were the number of spores and values of the Colony Forming Unit (CFU) in the *Aspergillus* sp. inoculum in corn media, with C, N, P, and P the C/N ratio of pineapple litter compost. The calculations of the number of spores and CFU values were conducted to determine the productivity and viability of the fungi inoculum, respectively. The compost content analysis was also performed in order to observe the quality of pineapple litter compost. The data obtained were analyzed descriptively and presented in graphical form. All the results were statistically analyzed using analysis of variance (ANOVA) test. Treatment means were compared using the least signiﬁcant difference (CD, P ≤ 0.05), which allowed the determination of signiﬁcance between different applications.

## 3. Results and discussion

### 3.1. The spore number of inoculum Aspergillus sp. in corn media

There is a relationship between nutrient complexity in the medium and the ability of fungi to grow and sporulate, which is also strain-dependent [25]. For the reproduction and growth of fungi to proceed properly, the microorganism required substrates containing nutrients for their metabolism. Corn has a high crude fiber content of 86.7%, consisting of 67%, 23%, and 0.1% of hemicellulose, cellulose, and lignin, respectively [26]. The high content of cellulose also supported the use of corn as growth media for cellulolytic fungi (*Aspergillus* sp.).

The results showed that the number of spores on the *Aspergillus* sp. inoculum was 5.64 x 10^7^ spores/mL, which was similar to the rate of the *A. parasiticus* species from previous studies, with similar media at pH 4–10, which was also in 10^7^ [27]. The best spore production in a previous study occurred at pH = 5, with the rate at 8.3 x 10^7^ cells/mL [27]. This was presumably due to the pH of the corn media being sufficiently in line with that of the optimum for fungal growth. Spore production was also influenced by environmental factors, including substrate, humidity, temperature, nutrition, and pH [28].

The increase in the number of spores occurred allegedly due to a suitable growth process due to aerobic metabolism. The large particle size of corn provided enough oxygen to have an impact on mycelial propagation, which in turn became easier [28],[29]. Also, available nutrients in the environment aid maximum mycelium growth until it reaches the logarithmic phase. Afterward, the nutrients are reduced, triggering the formation of spores as self-adaptation.

### 3.2. Viability of the Aspergillus sp. in corn media

The viability value of fungi *Aspergillus* sp. inoculum in the corn media obtained 4.4 x 10^7^ CFU/mL (Table 1). According to Mikata [30], isolates showing a high, moderate, low, and very reduced levels of viability, had mean CFU values ≥1 x 10^7^/mL, ≥1 x 10^6^/mL, around 1 x 10^5^/mL, and ≤1 x 10^4^/mL, respectively. Therefore, it was concluded that the viability of *Aspergillus* sp. in the medium of corn was at a moderate level. Also, *Aspergillus* sp. had a high life capacity. The inner membrane of fungal spores contained various enzymes and specific protein receptors, which were always quick to repair accumulated damages, while a thick layer protected the outer region.

Moreover, spores contain trehalose compounds, which regulate the osmotic pressure affecting the viability and tolerance of unfavorable environmental stresses. This result is in accordance with the result reported by Cazorla *et al*. which stated that the rate of conidia germination was determined by the content of the polyol and trehalose [31]. Also, spores play an essential role in the life cycle of fungi, which acts as a spread or survival. Another research by Garcia-Kirchner *et al*. [32] shows that corn containing media are suitable for Aspergillus growth. The research mentioned that *Aspergillus niger* is able to grow on corn containing media and shows its high lignocellulolisic activity, furthermore this corn serves as a carbon source to support its enzymatic activity.

**Table 1.** Average number of spores and viability of spore *Aspergillus* sp. inoculum in corn media.

Number of Spores (spores/mL)	Viability Value (CFU/mL)	Number of Spore Logs	Spore Viability Log	Percentage of Spore Viability (%)
5.645 x 10^7^	4.4 x 10^7^	7.75	7.64	98.58

### 3.3. Carbon content (C) of the pineapple litter compost

**Figure 1.**
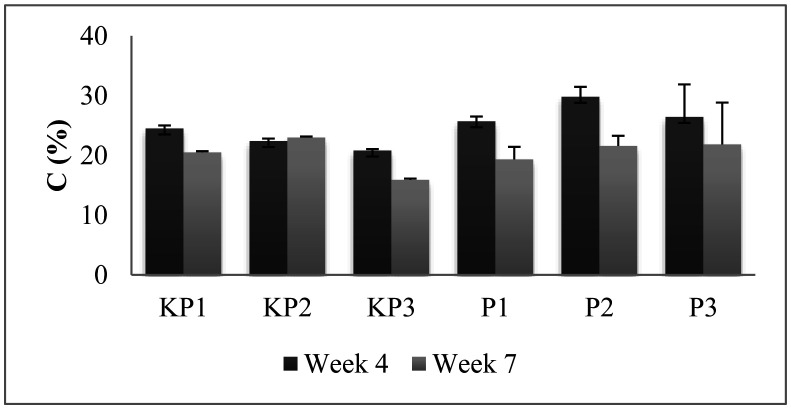
Carbon content of the pineapple litter compost. The test indicates there are no significant differences among treatments within the same week.

The data analysis shows there are no significant differences among treatments within the same week. However, the C level of pineapple litter compost in the 4^th^ to 7^th^ week tended to decrease. The sharpest decrease occurred in P2 (27.58%), with C levels in the 4^th^ and 7^th^ weeks at 29.8% and 21.58% (Figure 1). The activity of degradation and decomposition by microorganisms also caused a decrease in the compost material's C level. The result in this study was consistent with the result reported [33] which stated that C levels decreased according to the maturity of compost. This decrease was caused by the microorganisms' respiration and assimilation activities during the composting process [34]. Pineapple litter contains organic materials, which are energy sources for fungi, and are further converted into simple molecules. This observation was in agreement with the results of Suthar and Gairola, where this activity changed the available organic C, freeing it again in the form of CO_2_ gas [35]. During the composting process, the carbon elements will be released thereby reducing the amount of carbon. Some carbon- and nitrogen-containing gases are inevitably released during the process of composting due to the different operating conditions, resulting in carbon and nitrogen losses [36].

The absorption of nutrients by fungi was further carried out by remodeling the polymer substrate, using enzymes that were secreted into the environment. The previous report also showed that the genus fungi, *Aspergillus* sp., produced cellulase enzymes [37]. Cellulase enzymes degraded cellulose enzymatically and produced oligosaccharide and disaccharide compounds with soluble glucose monomers. Afterward, glucose was used as a carbon source for fungal metabolism. Glucose also provided maximum growth for fungi due to being more easily converted into phosphorylation of derivatives, which passes the pathway of the respiratory system [38]. The C content in pineapple litter decomposition fulfilled the Indonesian National Standard (SNI) 19-7030-2004 compost fertilizer quality standards, which is 9.8–32% [39].

### 3.4. Nitrogen (N) content of the pineapple litter compost

**Figure 2.**
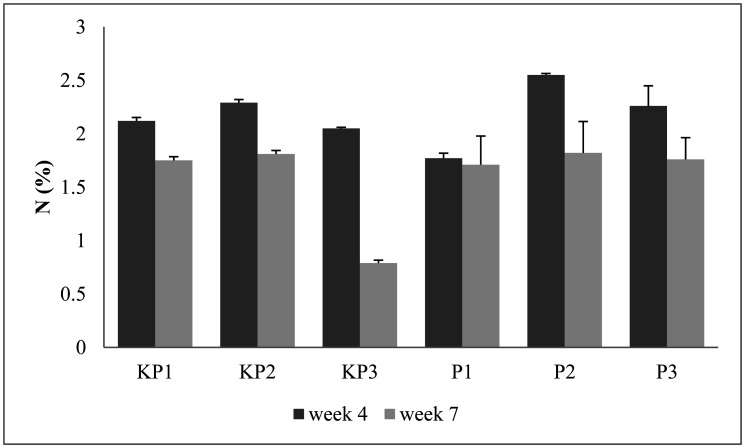
Nitrogen content of the pineapple litter compost. The test indicates there are no significant differences among treatments within the same week.

The data analysis shows there are no significant differences among treatments within the same week. However, the highest and lowest N contents of pineapple litter decomposition at 4^th^ week were discovered in P2 and PI composts at 2.55% and 1.77%, respectively (Figure 2). High levels of N were caused by the decomposition process of microorganisms that produced ammonia and nitrogen. However, the decrease in N levels was caused by the loss of nitrogen into the air, during compost mixing.

In the 7^th^ week, the highest and lowest N contents were obtained in P2 and KP3 composts at 1.82% and 0.79%, respectively. A decrease in N levels in the 7^th^ week indicated that composting time affected the percentage of nitrogen content. During the composting process, nitrogen mineralization decreased according to the time of fermentation [40]. It was further suspected that microbes still adjusted and metabolized in the initial phase, as their activities only increased cell size, with cells using carbon from the substrate as food and reproduction media for themselves. Afterward, the microorganisms reached an equilibrium amount between living and dead, therefore resulting in the declination of microbial activities, as indicated by the decrease in N levels in the 7^th^ week due to reduced carbon.

The general pattern of pineapple litter compost's N levels in the 4^th^ to 7^th^ week tended to decrease. The sharpest decrease occurred in KP3 (61.46%) (without inoculums), with N levels at the 4^th^ and 7^th^ week indicating 2.05% and 0.79%, respectively. The sharpest decrease occurred in P2 (28.62 %) in the inoculum applications, with N levels at the 4^th^ and 7^th^ week indicating 2.55% and 1.82%, respectively. The longer composting process caused nitrogen levels to decrease, due to the influence of cell metabolism, which resulted in N being assimilated and lost through volatilization (lost in free air), as ammonia [40],[41].

The final result of this mature compost shows promising results and still matches existing standards. Based on SNI: 19-7030-2004 regarding compost specifications of domestic organic waste, the minimum nitrogen yield was 0.4%, while the N contained in the composts was above the standard [36].

### 3.5. Phosphorus (P) content of the pineapple litter compost

**Figure 3.**
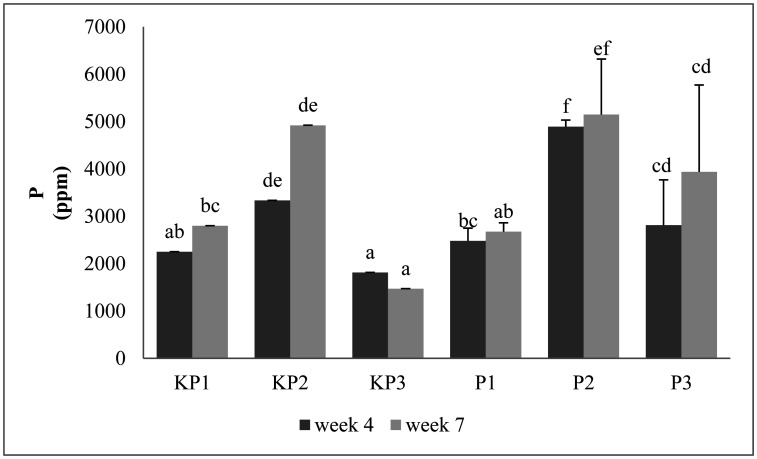
Phosphorus content of the pineapple litter compost. The same letter in the same weeks shows no significant difference among treatments.

Analysis data shows there are significant differences among treatments. The highest and lowest P levels of the pineapple litter in the 4^th^ week were obtained in the P2 and KP3 composts, at 4891.67 and 1813 ppm, respectively (Figure 3). KP3 compost was a treatment without the addition of an inoculum, as the activity of decomposing microorganisms was low, causing reduced phosphorus synthesis. However, the P2 compost was with the addition of an inoculum, which caused an increase in the number of microorganisms, and total P levels in the compost. In the 7^th^ week, the highest and lowest P levels were obtained in P2 and KP3 composts, at 5148.52 and 1468.71 ppm, respectively. Also, the treatment of KP3 in the 4^th^ and 7^th^ weeks decreased due to the composition of the compost leaving materials (mixture of tubers and pineapple leaves) without the addition of fungi inoculum activator, *Aspergillus* sp.

*Aspergillus* sp. inoculum affected the compost quality, as high levels of P were also influenced by an abundance of decomposing microorganisms. The more microorganisms, the quicker the maturity of the compost. Also, phosphorus in the matured stage of the compost was sucked up by the microorganisms. This was the reason phosphorus content in the 7^th^ week increased. [42] reinforced this statement by reporting that phosphorus increased with the number of microorganism cells. The increase in P-level was caused by the multiplicative presence of the microorganisms, which overhauled phosphorus and the process of mineralization by their existence in the formation of P [42]. This caused an accumulation of the phosphorus level contained in the raw material, and the number of microorganisms in the composting process [42],[43]. Fungi utilize phosphorus from the environment for metabolism, as their availability increases with increasing levels of P.

The general pattern of P content in pineapple litter compost tended to increase from the 4^th^ to the 7^th^ week. The sharpest increase occurred in KP2 (47.57%) (without inoculums), with P levels in the 4^th^ and 7^th^ week at 3334 and 4920.03 ppm, respectively. In the inoculum's treatment, the sharpest increase occurred in P3 (40.10%), with P levels in the 4^th^ and 7^th^ week at 2811 and 3937.26 ppm, respectively. The increase of P is presumably due to the decomposition process of organic materials occurred quite well in line with the increase of compost maturity. The pineapple litter compost's P level in the 4^th^ and 7^th^ weeks showed a significant difference. All compost variations were mature and stable while meeting the minimum P content following SNI 19-7030-2004, i.e., the total P-content was more than 0.1% [39].

### 3.6. Potassium (K) content of the pineapple litter compost

**Figure 4.**
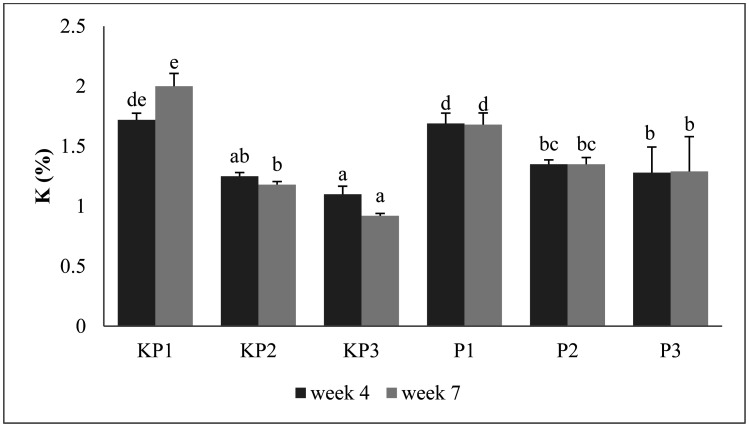
Potassium (K) content of the pineapple litter compost. The same letter in the same weeks shows no significant difference among treatments.

Analysis data shows there are significant differences among treatments. The highest and lowest K contents of pineapple litter composts at 4^th^ week were obtained in KP1 and KP3 composts, at 1.72% and 1.1%, respectively (Figure 4). In the 7^th^ week, the highest and lowest K contents were further obtained in KP1 and KP3 composts, at 2% and 0.92%, respectively. The KP3 compost had a lower stack height than other treatments. KP3 is a mixture of compost material with the most complex raw materials; thus, the decomposition of these mixtures was slower compared to the others; therefore, the K released was less.

Furthermore, *Aspergillus* sp. inoculum and composting time were observed to affect the K content of the compost. This was consistent with the opinion of Abubakar [27] which stated that the longer the stirring time, the lower the levels of potassium in fertilizers. This was due to the fact that the potassium bounded was released again. Also, the general pattern of the compost's K levels in the 4^th^ to 7^th^ week tended to be fluctuating. The sharpest decrease occurred in KP3 (16.36%) (without inoculums), with K level at the 4^th^ and the 7^th^ week had 1.1% and 0.92%, respectively. In the inoculums' application, the sharpest decrease occurred in P1 (0.59%) (without inoculums), with K level at the 4^th^ and the 7^th^ week had 1.69% and 1.68%, respectively. The rest observation shows an increasing pattern. This research was in line with other studies, which stated that potassium levels decreased due to being used as catalysts by microorganisms in the substrate material [34]. However, the K content of compost followed the minimum standard of SNI 19-7030-2004, which was 0.20% [39].

### 3.7. C/N ratio of the pineapple litter compost

**Figure 5.**
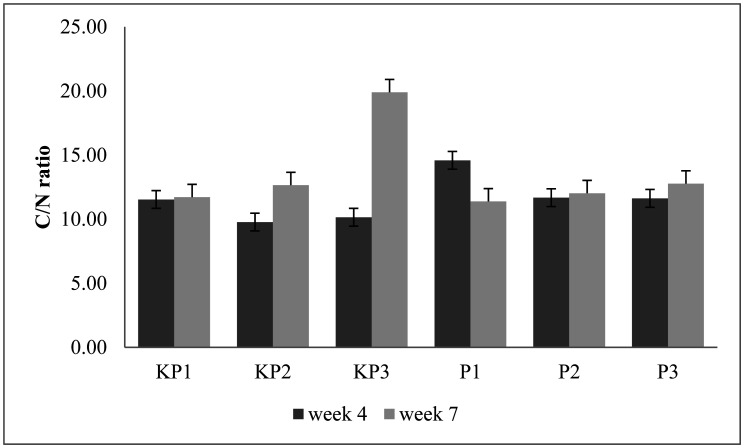
Compost C/N ratio of pineapple litter. The test indicates there are no significant differences among treatments within the same week.

The data analysis shows there are no significant differences among treatments. The highest and lowest C/N ratios of pineapple litter composts in 4^th^ week were obtained in P1 and KP3 composts, at 14.56 and 10.15, respectively (Figure 5). The high C/N ratio in the P1 compost was caused by the multiplication of microorganism cells using N levels in compost material, as a decrease does not follow it in C levels. However, the low C/N ratio in the KP3 compost was due to the high heap, as the degradation process acted quickly. The higher the pile, the smaller the porosity, causing the amount of heat generated during the decomposition process to be trapped inside the heap. It is also known that the pile height affected temperature, pH, and Moisture Content (MC), causing decreased microorganism activity [44],[45].

The C/N ratio in the KP3 compost was further influenced by the activity of microorganisms, as the decomposition process does not act optimally. Also, the higher C/N ratio indicated that the compost had not been completely decomposed [44]. Probably, the KP3 decomposed slower too than other compost substrates since it contains a more raw material combination. However, matured decomposed waste had physical characteristics changing the physicality of the compost [39], as the color became brown/black and odorless, while possessing crumb texture like soil [44]. Moreover, the compost's C/N ratio in the 4^th^ and 7^th^ weeks fluctuated (Figure 5). In the application of inoculums, the sharpest decrease occurred in P3 (16.36%), with C/N ratio level at the 4^th^ and the 7^th^ week had 11.61 and 10.23%, respectively. However, the value of the C/N ratio of pineapple litter compost P1, P2, and P3 was at the standard, according to the minimum technical requirements of solid organic fertilizer, which was 15–25 [45].

In general, the decomposition process of organic matter in nature involves almost all microorganisms with their respective niches. The Inoculant of *Aspergillus* sp. is important in this process because it plays a role in initiating the process of cellulose-cell decomposition (the majority in plant cell walls) making it easier for other microorganisms to dominate the next process.

## 4. Conclusions

*Aspergillus* sp. inoculum with enrichment of corn media can be used as an element to accelerate the pineapple biomass composting process. Also, the addition of *Aspergillus* sp. inoculum improved the quality of pineapple litter compost, as observed from the final contents of the decomposition process, in the form of C, N, P, K, and C/N ratio. Compost quality obtained was produced from an inoculum of 1% of pineapple substrate weight and possible to increase to achieve better compost yield. The application of 1% inoculum was able to mature the compost. It caused the sharpest decrease in C levels of 27.6% for pineapple stem litter and cow manure (2:1) compost materials, less effective for N and P changes. With cellulolytic inoculum addition, potassium and C/N ratio changes tend to fluctuate during 7^th^ week of pineapple biomass composting. In future reseach, a study will be developed on the inoculum characters under physical conditions (pH, temperature, salinity, humidity, pesticides) and biology (pathogenicity, mycotoxins, secondary metabolites) before field application.
